# Effects of learner control design in an AR-based exhibit on visitors’ museum learning

**DOI:** 10.1371/journal.pone.0274826

**Published:** 2022-10-18

**Authors:** Weijane Lin, Wen-Ting Lo, Hsiu-Ping Yueh

**Affiliations:** 1 Department of Library and Information Science, National Taiwan University, Taipei, Taiwan, R.O.C; 2 VIVE Research and Development Advanced Creative Team, HTC Corporation, Taoyuan City, Taiwan, R.O.C; 3 Department of Psychology, National Taiwan University, Taipei, Taiwan, R.O.C; 4 Department of Bio-Industry Communication and Development, National Taiwan University, Taipei, Taiwan, R.O.C; University at Buffalo - The State University of New York, UNITED STATES

## Abstract

This study shifts the focus away from demonstrating the existence of the effect toward understanding the mechanism by which the effect of AR operates in museum learning. By uncovering and describing the contingencies of AR from the perspectives of learner control, this study investigates how and when AR affects museum learning experiences, and to give insights into curation with AR. A between-subjects experiment was conducted with 48 college students divided into three groups. This study considered both qualitative and quantitative features of learner control and designed the AR control tools and experiment accordingly, and the findings supported the success of integrating the immersive technology of AR and the theoretical framework of learner control to construct museum exhibits. The results showed that visitors are willing to use the provided tools in museum AR and perform steadily in knowledge acquisition. In addition to offering more learner control in museums, AR promotes positive behaviors and attitudes. This study contributes to the field studies of learner control by linking learner control with the critical dimensions of AR-enhanced museum learning to provide more guidance in exhibit design. Based on the findings, practical suggestions on incorporating learner control in AR-based interactive exhibits are provided.

## Introduction

Museums are one of the main informal learning environments where people learn in a particular situation with real objects. As memory institutions, museums not only collect and display culturally meaningful artifacts but also affect visitors’ learning profoundly. Visitors achieve their learning by interpreting and memorizing incoming information during the visits on a more self-regulated basis with more control over the environment [1, 2]. As Falk and Dierking [1] noted, museum learning constructed from visitors’ experiences is highly related to the museum environment and exhibits. Later studies supported and proposed using multimedia to motivate visitors’ learning and engagement through increasing the realism of the environment [3, 4]. One simulation technology that offers an alternative way for museums to communicate with visitors in multiple forms of presentation and interaction is augmented reality (AR) [4–6]. Allowing intuitive interaction and manipulation of artifacts, AR facilitates visitors to maintain their attention on engaging with the surroundings, the exhibit display, and the materials [5, 7, 8]. However, despite the increased attention on using AR in museum exhibits, previous studies have mainly focused on the learning outcomes instead of the process of how AR affects visitors’ museum learning. Without knowledge of the underlying mechanism, it is difficult to develop design strategies based on the distributed and fragmental findings of the empirical works. Despite the positive findings of AR effects on presentation flexibility and visitors’ satisfaction [9–11], previous studies have also suggested that unknown factors might interfere with the interpretation of the AR effects [11–13], and further investigation will be required to uncover the relationship between AR design and museum learning.

Among the investigations of possible factors on learning effectiveness, studies in educational psychology suggest that the provision of learner control is positively related to learning outcome and process [14, 15]. Related studies in multimedia learning, though few are on AR, further echo the benefits of learner control on students’ engagement and performance [16–18] through more proactive interaction such as altering multiple representations and having multiple ways of interacting with the environment [14, 16, 19, 20]. When museum visitors develop their own experiences by interacting with the exhibits, AR can provide contextual feedback through multimodal channels, increasing the chances and quality of learner control, due to its nature of real-time integration and presentation [21, 22].

Motivated by the aforementioned issues, this study focused on how AR affects visitors’ museum learning from the perspective of learner control. This study aimed to investigate the effectiveness of an AR-based interactive exhibit designed with a theoretical framework of learner control [17] on visitors’ museum learning experiences. AR tools with different levels of learner control were first designed and developed, and then this study investigated how and when the visitors used these tools to achieve their learning in a museum so as to form a deeper understanding of the visitors’ experiences and performance. To compare learning experiences between visitors, given the different numbers of AR learner control tools, the subjects’ feelings of flow and learning performance and their behaviors were recorded for analysis. By exploring how visitors interacted with the AR learner control tools and their museum experiences, the study went beyond just reporting on learning outcomes, and it proposes suggestions on how to focus on learner control in AR design for improved museum learning experiences.

## Literature review

### Augmented reality in museum learning

The past decade has witnessed the tremendous growth of augmented reality technologies and applications. With the enhanced version of reality created by AR technology, which can overlay digital information on something being viewed [23, 24], AR applications in museums essentially consist of integrating digital content with a visitor’s sensory, usually visual, perception in order to perceive additional elements, thus augmenting a visitor’s space to enrich the real time experiences [4, 5, 11, 22]. Furthermore, AR offers the capability for users to artificially interact with the overlaid elements. Being able to physically interact with content that appears to be real has proved to be inspiring for museum visitors in terms of their learning experiences [22, 25, 26], and it also offers museums endless possibilities to engage their visitors. As major informal educational institutions, museums create authentic and meaningful learning experiences for visitors by displaying real objects and providing visitors with opportunities to interact with the exhibits [2]. In contrast to formal learning environments, museums, as supportive environments, rekindle visitors’ natural motivation to learn in a relatively concrete and realistic way [2, 27]. Visitors observe and reflect on the content with reference to their personal experiences in various experiential learning activities, such as their purposeful manipulation, active involvement, or transferal of ideas and skills [28]. Museums provide them with a wide range of situated tools to facilitate these activities [29–32].

Previous studies that used AR in both formal and informal educational settings have pointed out that AR combines real environments and virtual items, offers more delicate presentation, and promotes learning experiences and impressions [23, 33]. For presentation, AR performs well at displaying either abstract or invisible subjects, which helps learners to interpret and understand information better [26]. Attaching virtual information to reality not only supports visitors in understanding abstract knowledge but also presents more details about the exhibition [9, 11]. On the other hand, AR as a simulation technology imitates particular environments, objects or movements, and it enhances the sense of immersion and presence so as to involve learners through instant multimodal feedback [22, 25]. The interaction and interactivity enabled in AR environments facilitate learners’ situated learning [30, 34]. In addition to the learning outcomes and effectiveness, research attention on the learning process or mechanism with AR technologies has increased recently. Lu et al. [5] examined museum visitors’ experiences with AR from the media effect perspective. They compared the behaviors of art museum visitors within different exhibit media of AR and label text and found that AR was a better medium to attract and guide visitors’ attention. Hwang and his colleagues [35] adopted gamification strategies in AR-based ecology learning. They provided children with great autonomy in a competitive game to explore the surroundings and seek solutions to the game missions by using the available AR tools. Their findings suggested the need for instructional strategies in AR-based learning environments and also signified the importance of learners’ autonomy.

To understand visitors’ museum experiences in general and learning experiences in detail, researchers in related fields have adopted different approaches and measurements, including more interpretivist or qualitative paradigms in visitor studies, and the positivist or quantitative paradigm in educational research communities [36–40]. The idea of flow proposed by psychologist Csikszentmihalyi [41] is adopted to describe and measure visitors’ mental and physical engagement in their museum visits [37, 42]. Empirical investigations support that the state of flow facilitates visitors’ intrinsic motivation, results in better knowledge acquisition, and increases active learning behaviors such as concentration on and interaction with the exhibit content [43–45]. On the other hand, visitors achieve a state of flow in many different ways. Some rely on multimodal channels to enhance the sense of presence [44, 46]. Others are attracted and fascinated by the exhibit theme [47], while still others are motivated by a sense of pilgrimage in visiting certain museums or heritage sites [27, 48, 49]. The flow experiences not only affect active behaviors but also make people feel as if they are experiencing the activity in person. Latham [50] speculates that visitors who experience flow can relate the content of the learning in detail. Therefore, the museum learning experience consists of a learning process and its effect. The former shows the flow of visitors in the process of learning and engaging in positive behaviors, while the latter reflects learning performance and attitudes towards the learning experience and subjects.

### Learner control and AR-based learning environment

Learners’ interactions within the AR environment are highly involved with their autonomy and control over the learning situation. All interactions need to be actively triggered and linked by the users, making AR a highly user-controlled environment. In instructional technology studies, the idea of learner control refers to design features of the instructional interface or content that enable learners to autonomously decide and choose the path, rate and feedback while learning [51, 52]. Generally, previous investigations in learner control design have supported that being in control of learning leads to greater achievement by the learners [16, 17, 53]. These studies also pointed out that the provision of control tools is beneficial to knowledge acquisition, attitude and learning behaviors. These tools promote intrinsic motivation because the learner can act without restraint [18, 54]. On the other hand, while most studies have focused on the interface design of control tools as investigations of functionality [52], Lawless and Brown [17] focused on learners’ interactions with the content and divided learner control into different levels: browsing, searching, connecting, collecting and generative. They valued the qualitative nature of learner control revealed in learners’ behaviors and intentions. Their findings suggested that in multimedia learning environments with lower control levels, learners tended to gain a superficial understanding through quick and casual observation, regardless of whether they were just wandering around or had a specific searching goal in mind. When empowered to control the learning content, however, the learners were able to establish relationships between materials and even contribute new items to the learning environment. The perspectives and findings from Lawless and Brown [17] have significant implications for designing learner control because human interaction and processing strategies for content are rather constant and stable across different technological features.

Given the essence of learner control as a design feature of the learning environment, it is therefore closely related to the technology used [52]. Compared with screen interaction, AR technologies in multimodal presentation allowing direct manipulation may increase and enhance the forms and flexibility of learner control. First, in terms of presentation, AR leverages the 360 space by augmenting the real world with virtual information and therefore preserves sufficient user schema of interacting with external stimuli, resulting in more intuitive and autonomous actions. Learners in an AR-based learning environment are not only empowered to attend, arrange and manipulate the existing content directly but also encouraged to create new information and test different ideas. In previous studies that blended three-dimensional virtual objects into the real world to display different views, it was found that the learners were encouraged to observe and manipulate the objects from different angles [23, 33]. Second, AR extends reality by incorporating and integrating temporal and spatial information. Historical artifacts or heritage items that have been destroyed or lost over time can be recreated with AR [49, 55]. Finally, AR provides users instant feedback in response to their actions, enhancing participants’ sense of presence and willingness to interact with the environment [26].

With reference to the abovementioned features of both the instructional interface and content for designing learner control, this study adopted AR technology as the platform to construct a museum exhibit and provided various tools for interacting with the physical context, including the artifacts, content and environment, to achieve different levels of learner control. By employing a qualitatively and quantitatively different learner control design, this study investigated the impact of the AR tools on participants’ museum experiences by examining their flow state, learning performance and visiting behaviors.

## AR-based interactive exhibit with learner control

**Fig 1:**
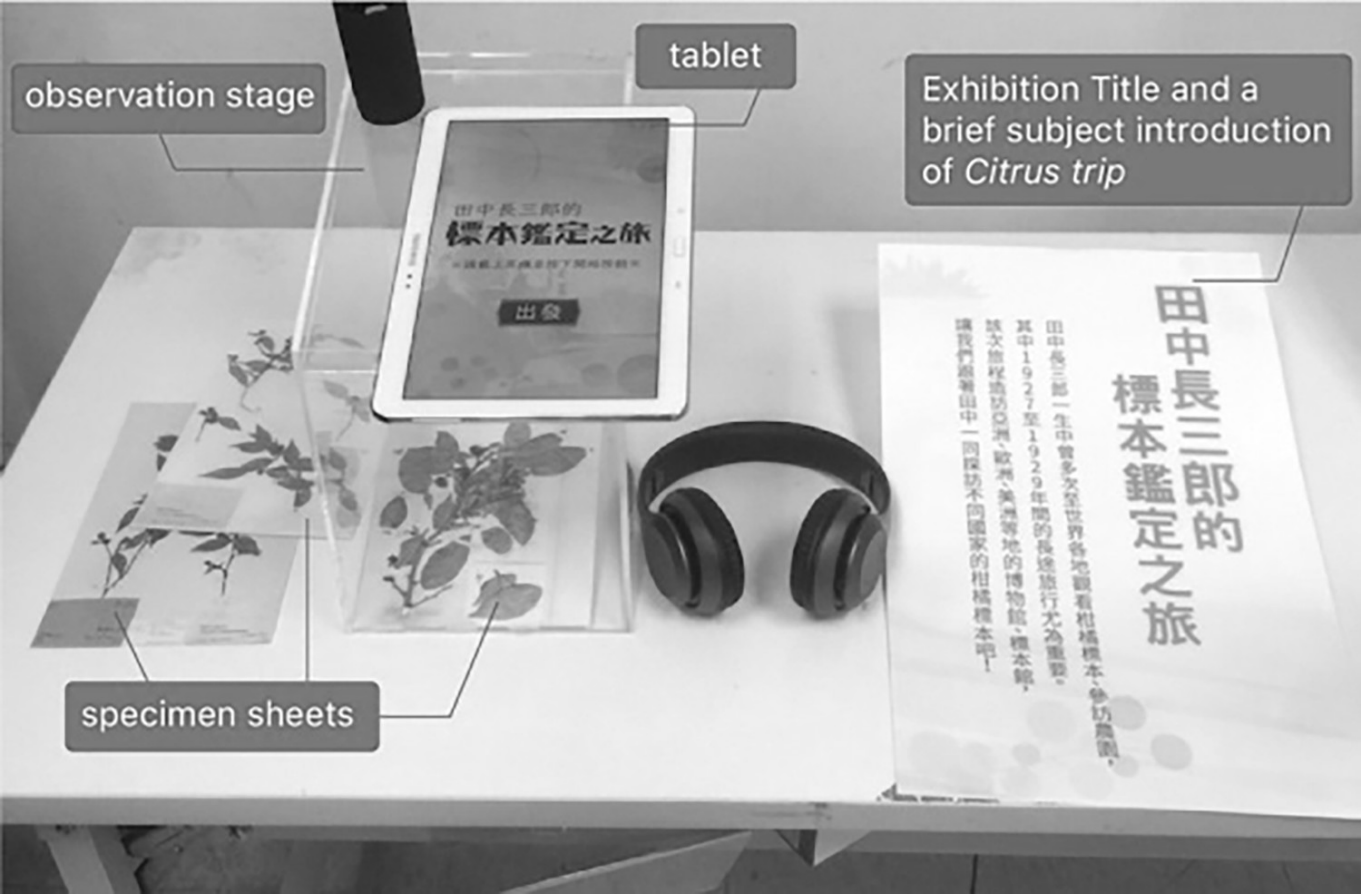
The experiment scenario. Note: The authors created this figure for this article; it is not based on any previously copyrighted image.

To discover how learner control affects the museum learning experience, an AR-based interactive exhibit of a special collection of herbarium specimens was designed to provide different levels of learner control. The exhibit was technically developed with augmented reality technology, and the exhibit content was designed and presented as a story that incorporated a scenario, narrative and interactive elements to provide learners with sufficient environmental context [1, 24]. The exhibit, entitled “Tanaka’s Journey of Citrus Fruit Specimen Identification”, displayed a special collection of citrus fruit specimens housed by the University Museum. By using recognition-based AR technology with video see-through display on a tablet computer, this exhibit conveyed the thematic knowledge and skills related to herbarium specimens, while also allowing the visitors to experience the heritage valorization of this special collection through interactive storytelling. The content of the exhibit covered a range of topics, from the origin of the collection [56], presented by showing how the famous taxonomical botanist Tyozaburo Tanaka collected the large number of citrus fruit specimens around the world in the early 20th century, to the procedures of identifying each and every specimen in the field. This exhibit simulated the contexts, tools and tasks for the participants to see, hear, and handle the herbarium specimens. The designed AR system was displayed in an experimental gallery on the university campus, and an overall image of the exhibit is shown in Fig 1. During their visits, museum visitors were allowed to pick up the physical specimens and observe them with the tablet.

**Fig 2:**
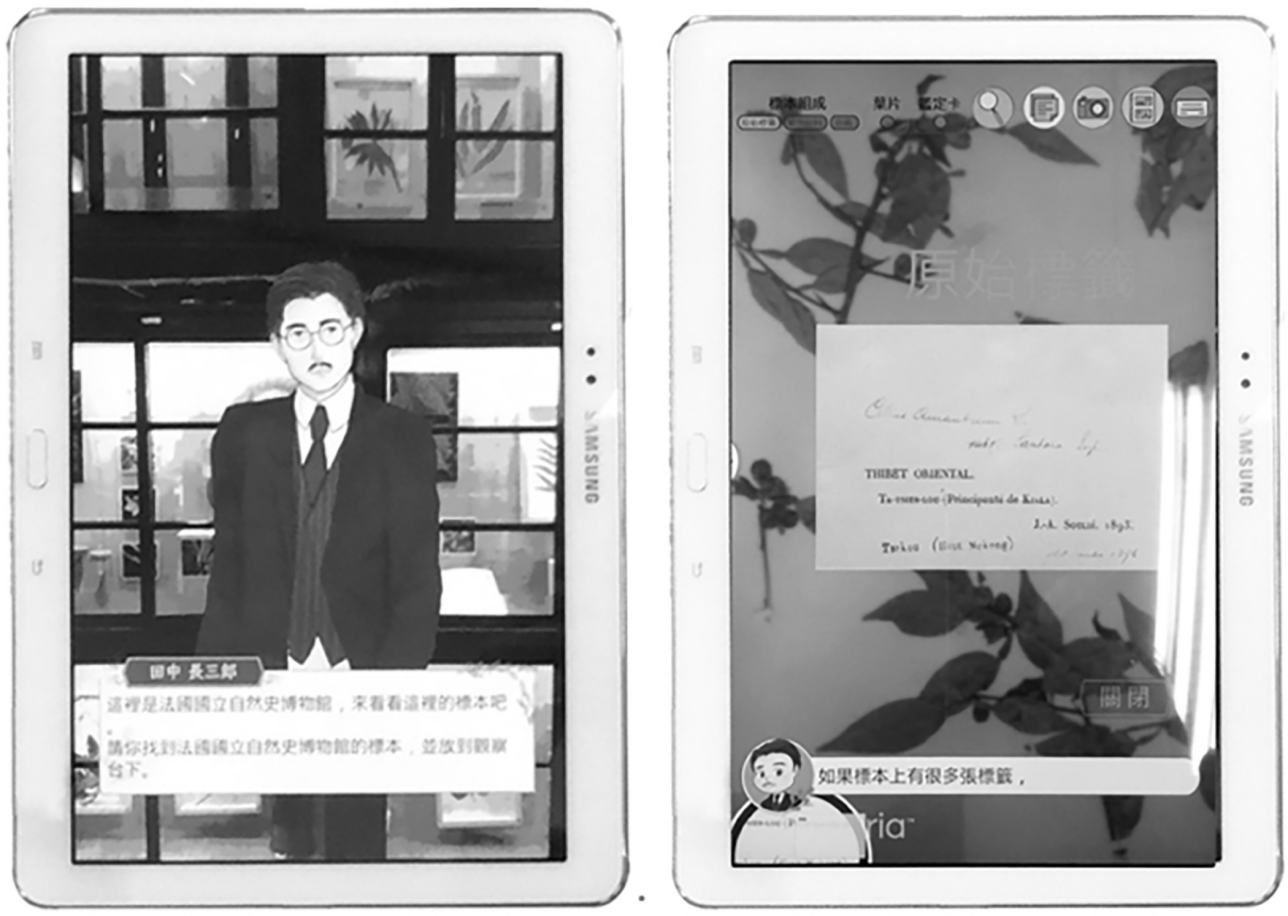
User view of the AR exhibit in a real-world context. Note: The authors created this figure for this article; it is not based on any previously copyrighted image. The authors created the images on the screen of the tablet in this figure.

This study followed general principles of constructivist theory and multimedia learning [31, 32] to structure and segment the multimodal materials. As shown in Fig 2, when a visitor pressed “Start” on the tablet to start visiting the interactive exhibit, the taxonomical botanist Professor Tanaka, the main character of the story, would appear and greet the visitor with a brief introduction. Within the AR-based exhibit, all the physical objects, including the specimens, tablet computer, and participant, were registered and calibrated by the system in order to provide real-time feedback that corresponded to the user’s actions. As shown in Fig 2, all the paper images and physical objects were predefined for cameras to easily recognize and process. When a visitor scanned the specimen by moving it under the tablet camera, the augmented experience would be triggered to present an enlarged picture on the tablet screen to notify viewers of the content they were viewing, and relevant information in different modalities would be rendered and presented in the forms of narration, text, and graphics.

To investigate whether different levels of learner control affect museum learning experiences, this study designed and developed 4 types of AR tools, with reference to Lawless and Brown [17]. As shown in Table 1, the four tools were the Magnifier, Camera, Album and Notebook, which represented different levels of learner control for interacting with the content. Additionally, these control tools were situated tools that accommodated the learners’ activities of specimen identification in the field. The Magnifier was designed for browsing and connecting to the presented materials. The Camera and Album were designed for users to collect available information for recital, and Notebook was designed as more advanced form of learner control over the content to enhance generative learning by facilitating learners’ cognitive strategies of content elaboration and organization. In addition to the types of tools, we also provided different levels of control within a single tool, the Magnifier, to investigate the qualitative differences among learners’ initiatives. There were three kinds of Magnifiers, which allowed learners to reactively enlarge the prompt content (A1), actively move around the prompt content (A2), and proactively observe anywhere on the screen (A3).

**Table 1:** The design of the AR learner control tools.

Tools	Type and definition		Learner Control
Magnifier	A1	Trigger the four sets of learning content	browsing connecting
	A2	Move randomly to trigger learning content	
	A3	Determine which learning content to see	
Camera	B	Record learning content in image immediately	collecting
Album	C	Browse pictures taken by camera tool; enlarge pictures to see more details	collecting
Notebook	D	Write and place text records on specimens	generative

In addition to the qualitative nature of the control level, this study further investigated the quantity of learner control and developed three versions of the exhibit with different numbers of AR learner control tools. As shown in Table 2, the Low control version consisted of only the reactive Magnifier (A1), the most limited learner control. The Medium control version allowed more learner control in connecting and collecting content information with the active Magnifier (A2), Camera (B), and Album (C). The High control version provided users with four types of learner control tools, namely, the proactive Magnifier (A3), Camera (B), Album (C) and Notebook (D), which allowed learners to freely browse, connect to, collect and generate content.

**Table 2:** Three versions of exhibit with different number of AR learner control tools.

	Auxiliary Functions			
	browsing	connecting	collecting	generative
Low control	A1			
Medium control	A1	A2	B, C	
High control	A1	A2, A3	B, C	D

With these AR learner control tools in hand, visitors were guided by Professor Tanaka to use the tools to observe and record the information about the specimens during their visits. For example, in the task of observing the specimen, Professor Tanaka would suggest that the visitor imitate him in using the AR tools to accomplish four learning activities, from observing a whole specimen to noticing the specific features of citrus fruits. The learning units were namely (1) observing the materials of the setting paper, (2) looking carefully at the components of the herbarium specimen, (3) reading the information on the original identification cards, and (4) observing the petiole wings of the plant. After the observation task, Professor Tanaka would present a situated quiz and ask the participant to match the correct specimens and identification cards.

## Experiment design

### Participants

A total of 48 college students voluntarily participated in the experiment. Their prior knowledge and experiences of the museum, augmented reality technology, and herbarium specimens were investigated in advance, and the participants were divided and assigned into three groups to experience the three different versions of the AR exhibit. The Research Ethics Committee of the University approved all procedures, the protocol, and the methodology (NTU-REC 201807HS006). All participants’ signed written consent forms were obtained before the experiment.

### Instruments

This study adopted quantitative and qualitative measurements with the Personal Context Questionnaire, the Flow State Scale, the Performance Test, and the Behavior Mapping Log to investigate participants’ museum experiences, learning processes and outcomes in the AR-based interactive exhibit.

The Personal Context Questionnaire was distributed before the experiment to investigate participants’ personal contexts, including their prior experiences with the museum, AR, and herbarium specimens. Prior experience included four aspects: awareness, reaching frequency, purpose, and familiarity. Each aspect was transformed into a score, and the total score was calculated to represent the personal context of each participant.

As a measurement to understand the constructs of participants’ flow experiences, the Flow State Scale (FSS) proposed by Jackson and Marsh [57] was adopted. Despite the difficulty of capturing flow with a single instrument or method, Jackson and Marsh’s measurement of distinct components of flow provides a better basis for evaluating the theoretical underpinnings of flow [41] than does reliance on a global score. The FSS consists of 36 items that measure nine constructs of flow experiences, as follows: the clarity of goals, challenge–skill balance, action–awareness merging, unambiguous feedback, complete concentration on task at hand, feeling of control, loss of self-conscuousness, transformation of time, and the autotelic experience. The questions were translated into Chinese with reference to the original descriptions of the constructs and items [44]. For example, one of the questions on participants’ clarity of goals was “I know exactly what I want to see/do in this exhibition.” To investigate the sense of control, the question “I feel like I have complete control over what I am doing” was used, and the question “I enjoyed myself and I am not worried about what people think of me” was used to investigate their autotelic experiences.

Participants’ learning outcomes were assessed by a multiple-choice quiz consisting of measurements of memory and comprehension performance [32]. The Performance Test consisted of 8 items, of which 6 were memory questions to investigate the extent to which participants recalled the information, and 2 were comprehension questions to understand if they could engage in application and problem solving based on their learning. Participants received 2 points for each question for correct answers, 1 point for partial correctness, and 0 for wrong answers.

To prevent interference, the observation of visitors’ behaviors during the experiment was conducted remotely through video monitoring by two researchers outside of the experimental galley. A Behavior Mapping Log was composed with reference to Griffin’s [28] indicators of museum learning behaviors, along with the temporal and spatial records of the participants’ dwelling times, positions, and uses of the learner control tools. After participants finished their visits, interviews were conducted for comparison of their subjective feelings and reflections with the data collected by the instruments.

### Procedures

**Fig 3:**
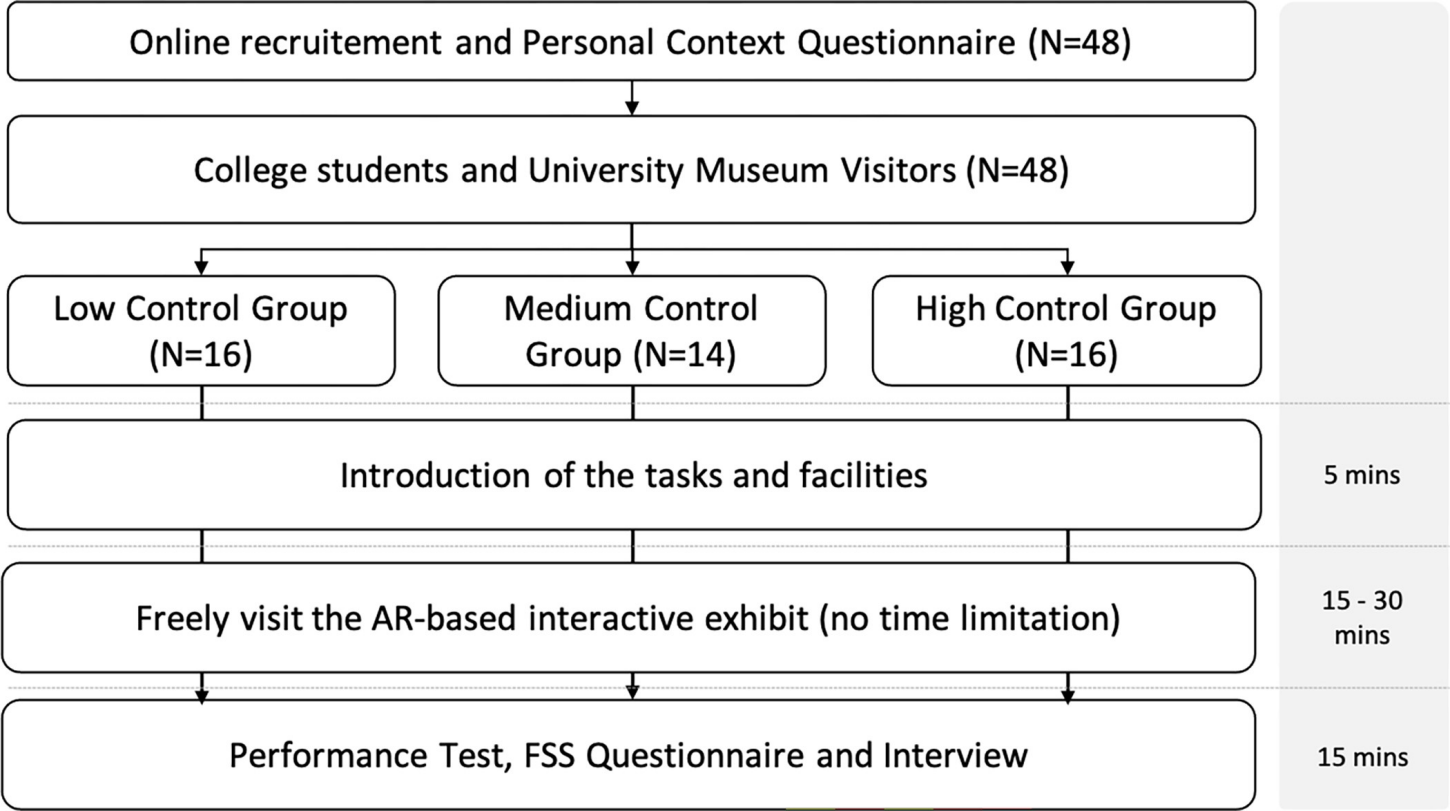
Procedure of the experiment.

Fig 3 illustrates the procedures of the experiment. Participants’ prior experiences were investigated during the online recruitment with the Personal Context Questionnaire, based on which the participants with different amounts of experience were evenly assigned into the three groups. Participants were invited to the experimental gallery and told beforehand that they could visit the exhibit as they normally would in a museum.

During the participants’ visits, the researchers used a video camera and two 360-degree cameras to remotely monitor the environment as well as the participants’ interactions with the AR. Participants signified their completion of the visit by leaving the gallery, at which point they were invited to another room to take the Performance Test and reflect on their visits using the Flow State Scale. A follow-up interview was conducted at the end to investigate participants’ subjective perceptions and feelings about the exhibit and their experiences.

## Results & discussion

### Demographics data, prior experiences of museum and AR

In all, 48 subjects were recruited to participate in the experiment, including 15 males and 33 females aged from 18 to 45 years old (M = 24.26, SD = 4.13). Based on the responses in the pre-test Personal Context Questionnaire, subjects with different backgrounds and prior knowledge were equally assigned to the three groups such that all had similar compositions. Two participants in the Medium Control Group dropped out of the experiment at the end, and their data were therefore excluded from the analysis. As shown in Table 3, all 46 participants were college students and had been to the University Museum. They visited libraries and museums for research or study needs to complete class assignments or projects. They were familiar with herbarium specimens and had actually worked with specimens in secondary school. Compared to the thematic knowledge and experiences, participants were less familiar with AR technology. They reported that they had a general idea of what AR was, although not many of them had actually used it before.

**Table 3:** Prior experience of participants (N = 46).

Prior experiences	Mean	SD
Libraries and museums visiting experience	6.74/11	1.95
AR experience	2.91/5	1.71
Herbarium specimen experience	2.54/3	0.86

### Flow experience

Flow experience, measured with the FSS, showed that average scores of the three groups of participants were all above 4.00/6. The global scores of the flow experiences indicated whether the participants had fewer tools (M = 5.10/6; SD = .632), had a moderate number of tools (M = 5.11/6; SD = .604) or had the most abundant tools (M = 4.79/6; SD = .723), they all experienced the state of flow when using the AR tools. This finding is consistent with previous studies that illustrated the advantages of AR in improving participants’ engagement in especially the hands-on interaction with the physical objects because of the integrated virtual and real-world representations [58]. Despite the perceived difference between the lower and higher control groups, as shown in Table 4, the difference among the three groups in all constructs suggested no statistical significance. While this result could have been affected by the small sample size, this finding was also consistent with those of previous studies [4, 43], as the experiment session was possibly too brief for the participants to actualize the flow state.

**Table 4:** Comparison of participants’ flow experience across groups.

Constructs	Group	N	M	SD	F value	p value
Clear Goals	Low control	16	5.19	.834	.959	.391
	Medium control	14	5.21	.893		
	High control	16	4.81	.981		
Challenge-Skill Balance	Low control	16	5.13	.806	.849	.435
	Medium control	14	5.00	1.038		
	High control	16	4.88	1.025		
Action-Awareness Merging	Low control	16	4.88	1.025	.849	.435
	Medium control	14	4.64	1.008		
	High control	16	4.38	1.204		
Concentration	Low control	16	5.06	.854	.886	.420
	Medium control	14	5.43	.756		
	High control	16	5.19	.655		
Sense of Control	Low control	16	5.06	.854	2.707	.078
	Medium control	14	5.29	.914		
	High control	16	4.50	1.095		
Loss of self- consciousness	Low control	16	5.19	.834	1.512	.232
	Medium control	14	5.36	.745		
	High control	16	4.75	1.291		
Transformation of Time	Low control	16	4.88	.957	.532	.591
	Medium control	14	4.50	1.286		
	High control	16	4.50	1.265		
Unambiguous Feedback	Low control	16	4.88	1.025	.427	.655
	Medium control	14	5.14	.864		
	High control	16	4.88	.806		
Autotelic Experiences	Low control	16	5.69	.479	1.394	.259
	Medium control	14	5.43	.938		
	High control	16	5.25	.775		
Overall flow experience	Low control	16	5.10	.632	1.204	.310
	Medium control	14	5.11	.604		
	High control	16	4.79	.723		

However, the qualitative investigation with the triangulation of interviews and behavior logs, focused on the perceptions of the tool interactivity of the participants, showed that the participants’ visit experiences were very different among the three groups in terms of the flow constructs. 12 subjects in the high control group and 9 in the medium group reported that they perceived a greater sense of control by actually manipulating the virtual tools to interact with the physical objects of specimens during their visits: “*I just followed my inclinations*. *I watched and did what I liked*. *It was fun to choose the positions of the magnifier and the scanning specimen*” (H-5). “*Tools and operations made me feel I was operating the real equipment*” (H-8). “*I liked using these tools when operating the magnifier*. *It provided instant feedback that allowed me to engage when using it*” (H-9).

In contrast, 10 of the 16 participants in the low control group pointed out that they were distracted by their inability to operate the AR tools autonomously. Many of them felt frustrated when they realized that they had little control over the learning process and AR content in the first few attempts. They also reported their inability and distraction in catching up with the program control. “*All I could do was put the specimen under the observation desk and start playing with the magnifier*. *I spent most of the time listening to the narration*, *so it was difficult for me to concentrate on the AR*” (L-14). Participants also felt powerless due to poor action–awareness merging [17, 45] when they pressed different buttons or touched any area on the screen and received no feedback. “*I could not decide what to look at or how many times to repeat it*. *I don’t think I was controlling the tablet*, *AR or content*. *It did not feel like observing the specimen and it distracted me*” (L-9).

The results of the interviews revealed two major factors that affected the participants’ flow experiences. First, more interaction with the exhibit content provided the participants with more immediate and clear feedback and resulted in better flow experiences. Eleven participants in the low control group complained about being distracted from the visit because there was no interaction. Another 8 participants in the medium and high control groups said that the interaction increased their concentration and pleasure of experiencing the exhibit. “*I could handle these tools*, *and I felt I was interacting with AR*. *What it showed told me what I should do at the moment*, *and these many tools I could use also increased my engagement*” (M-16). “*The way to move the magnifier to a specific position and reach the instruction was new to me*. *It made the learning content less boring*. *I felt I was interacting with the content*” (H-13).

Second, the context-specific operation of AR control tools positively affected users’ flow experiences by enhancing physical immersion. This finding is consistent with previous studies of immersive technology, wherein the participants experienced the virtual tools as actual physical equipment and enhanced their physical presence accordingly [59]. The design of the scenario, which simulated a taxonomical botanist’s work of identifying specimens, was proved to be successful in facilitating participants’ engagement and learning. “*I felt I was observing a specimen in the real field when I used the magnifier to zoom in; everything became so real*. *(The specimen) was no longer just something hanging on the wall*” (M-7).

In addition, not only the number of tools but the contextual ways the tools were used affected participants’ flow experiences. Taking the specimen identification card as an example, participants within the low control group had no control over the tool. They could only see how the character had used the tool on the display with narration. They also reported more frequently that the tool interrupted, rather than facilitated, their visits. Participants who were given a higher level of learner control with more tools and interaction, on the other hand, reflected a more plausible illusion and tended to be more engaged. “*The process of attaching the identification card was similar to what was done by Professor Tanaka*, *so I could imagine the situation of specimen identification*” (M-14). “*When I attached the identification card to the specimen*, *I suddenly realized that this was what Professor Tanaka had done decades ago*” (H-9). This finding is consistent with previous studies of learner control, as active control aids participants’ acquisition of the abstract concepts of the rationale behind specimen identification [60].

Both the quantitative and qualitative data showed how the participants positively engaged in the AR exhibit with different levels of learner control. The results suggested that the number, affordance and context of the learner control tools affected participants’ flow experiences. It was worthy of note that the affordance of the learner control tools was seen by the participants as more important than the availability of more tools. They valued the responses of the learner control tools to their situated cognition and actions in the context.

### Learning outcomes and processes

Participants’ learning outcomes were divided into two aspects, namely, memory and comprehension, and measured by the Performance Test. The results showed that participants within higher learner control groups had better performance in both memory and comprehension, but the differences were not statistically significant (see Table 5). This finding is consistent with previous studies of museum learning, as the visitors did not place as much emphasis on mastery of learning as they did on school education, and their learning outcomes could be manifold, involving knowledge, skills, attitudes, enjoyment and progression [2, 40]. Therefore, the qualitative inquiry was further conducted to capture the participants’ processes of learning.

**Table 5:** Differences of participants’ learning performance among groups.

Factors	Group	N	M	SD	F	p
Memory performance	Low control	16	6.63	.437	1.198	.312
	Medium control	14	7.57	.562		
	High control	16	7.50	.465		
Comprehension performance	Low control	16	2.19	.209	.703	.501
	Medium control	14	2.00	.277		
	High control	16	2.44	.288		

While the statistical test did not reveal significance, the qualitative analysis of data on participants’ learning behaviors and processes supported a positive association between learner control and generic learning performance. First of all, participants within higher learner control groups spent significantly more time on their visits (see Table 6). Spending more time on visits was seen as an active museum learning behavior [1, 2, 40], and this finding also echoed those of previous studies reporting better flow experiences [41, 42]. Participants felt encouraged to explore the exhibit content in their own ways with a variety of control tools whenever they needed them. Therefore, they spent more time trying out possibilities and became more engaged in the exhibit.

**Table 6:** Difference among the dwelling time of the three groups.

Group	N	M	SD	F	p	Dunnett’sT3
						post hoc test
Low control	16	382.52	123.04	13.004	.000**	High>Low
Medium control	14	450.16	172.39			
						High>Medium
High control	16	825.83	397.31			

**p < .01.

The behavior logs of participants’ viewing times also suggested similar findings. Participants with more learner control tools tended to interact more actively with the AR exhibit. Table 7 showed the frequencies of the participants’ viewing of the four learning units of specimen identification, from observing the whole specimen to examining the detailed features of citrus fruits. Participants within the low control group only clicked and played the content in order. Those in the medium control group responded to the visual cues on the screen and clicked the corresponding tools to proceed according to their own wills. Participants in the high control groups who viewed the content by themselves spent almost three times the amount of time on every learning unit. They tried out different ways of viewing, cross-referenced between the narrations and the specimen, switched to other control tools, and took notes.

**Table 7:** Frequencies of visiting different learning units across groups.

Learning Units	Group	N	M	SD	F	p	Dunnett’s T3
							post hoc test
(1)	Low	16	1.81	0.54	10.107	.000**	High > Low, High > Medium
	Medium	14	1.36	0.63			
	High	16	3.44	2.22			
(2)	Low	16	1.81	0.54	1.930	.158	
	Medium	14	1.93	0.83			
	High	16	1.94	1.39			
(3)	Low	16	1.81	0.54	6.390	.004**	High > Low, High > Medium
	Medium	14	1.43	0.65			
	High	16	3.00	2.16			
(4)	Low	16	1.81	0.54	10.512	.000**	High > Low, High > Medium
	Medium	14	1.57	0.76			
	High	16	3.81	2.48			

**p < .01.

Note: The number of the Learning Units refers to (1) observing the materials of the setting paper, (2) looking carefully at the components of the herbarium specimen, (3) reading the information on the original identification cards, and (4) observing the petiole wings of the plant.

### Uses of AR learner control tools

Generally, the participants were aware of whatever was available due to the guidance on the screen provided by AR, but they paid special attention to and actively used the Magnifier and Camera, which provided immediate and direct feedback to accomplish basic interaction, and controls like browsing and connecting to the content. Some participants were rather unfamiliar with the exhibit theme of herbarium specimens. Most of these relied heavily on the physical context for guidance to develop a general understanding. As shown in Table 8, the participants in the three groups all noticed and opened the AR tools provided on the tablet screen as possible external cues, but they did not use the tools as frequently or repeatedly for functions that required more advanced interaction with the content, such as zooming in on details of photos in the Album to make sense of all the collected materials or checking again what they had recorded in Notebook for generative learning. A noteworthy result from the triangulation of observation and interview data was that participants in the higher control groups had clearer goals in using each tool. They were intuitive in choosing the control tools to adapt to the tasks and challenges in the context, while the participants in the low control group tended to regard the control tools as a disturbance. “*The system just showed a Magnifier and kept explaining*. *I was distracted because I could not handle what I was doing*” (L-13).

**Table 8:** Number and frequency of AR tool usage.

Group	Tool		# of users	% of use	Frequency			
					M	SD	Min	Max
Low control	Magnifier		16/16	100%	1.81	0.54	1	3
Medium control	Magnifier		14/14	100%	9.21	3.33	4	14
	Camera		13/14	92.8%	3.31	2.63	1	9
	Album	open	12/14	85.7%	1.75	1.22	1	5
		enlarge	4/14	28.5%	2.25	1.50	1	4
High control	Magnifier		16/16	100%	30.56	14.18	13	59
	Camera		13/16	81.2%	2.61	2.43	1	9
	Album	open	15/16	93.7%	1.93	1.57	1	6
		enlarge	3/16	18.7%	2.00	1.00	1	3
	Notebook	open	15/16	93.7%	3.27	2.74	1	8
		create	10/16	62.5%	1.60	0.97	1	4
		check	8/16	50.0%	2.13	2.23	1	7

It was also found that the participants used the control tools as an external cognition mechanism to relieve their cognitive load and help them process the content. Over half (62.5%; 10/16) of the participants in the high control group took notes about the narration, the specimen, and even their own reflections. For example, one participant reported “*When the exhibition content was very long*, *I preferred to write down keywords in the Notebook*. *It reminded me of important things*” (H-13).

Interviews with the participants indicated that those who possessed higher learner control were more highly motivated to learn and explore the unfamiliar content with the facilitation of the AR control tools. When a participant in the medium control group followed the narration and used the Magnifier to look closely at the petiole wings of the citrus fruit, she spontaneously picked up another specimen sheet for comparison. “*I was very curious about these tools*. *They looked interesting*, *so I tried to use them*. *I felt like I was playing a game*, *and I clicked these buttons spontaneously*” (M-11). On the other hand, participants used the control tools mainly for tasks of reciting and summarizing. “*I immediately took photos and recorded important scenes*. *It helped me understand and learn*” (H-11).

Further examination of the amounts of time the participants spent using each tool (see Table 9) revealed an association between learner control forms and their museum experiences. The results revealed two important mechanisms to facilitate participants’ visits and engagement. First, as the participants tried more of these tools at different times or for the various tasks, they noticed that the tool functions responded to their situated cognition and actions. This responsivity motivated them to try and use the tools. Second, the timing and interaction provided by the tools greatly affected the participants’ willingness and effectiveness in using them in their visits. The Magnifier is a prime example. In the low and medium control groups, whose visits were more guided by the program, the Magnifier could trigger limited areas and interactions, and the participants spent similar amounts of time using the tool. However, in the high control group, the participants were liberated with the greatest learner control over the pace, content and use, and they used the Magnifier much more often and for longer periods of time according to their own needs and intuition. Participants in the high control group specifically mentioned a sense of control and realism obtained by using the Magnifier. “*This was not just listening to what Professor Tanaka said when using the Magnifier*. *I could actually play by myself*” (H-2). “*I focused on using the Magnifier and liked to find out what I hadn’t observed*. *I was eager to know what else I could see and why I couldn’t complete the task*” (H-1).

**Table 9:** Time spent using each AR tool in the three groups.

Group	Tool		# of users	Time spending (secs)			
				M	SD	Min	Max
Low control	Magnifier		16	386.49	133.16	226.55	521.17
Medium control	Magnifier		14	302.03	114.80	183.74	529.24
	Camera		13				
	Album	open	12	27.01	55.26	1.10	198.42
		enlarge	4	10.25	11.60	2.15	27.32
High control	Magnifier		16	604.97	261.52	267.02	1088.61
	Camera		13				
	Album	open	15	11.99	14.67	1.18	179.84
		enlarge	3	5.93	5.47	1.20	17.80
	Notebook	open	15	50.94	67.77	1.53	261.14
		create	10	1.60	.97	1	4
		check	8	3.14	3.62	.59	11.60

Combining quantitative and qualitative findings, it was evident that the multimodal presentation and immediate feedback provided by augmented reality technologies facilitated the learners’ control over the exhibit content and context, thereby enhancing their museum experiences. All participants, regardless of group, were able to achieve the state of flow in the AR environment. In addition, the control tools and features enabled control over pacing, content and access to learning support, in turn providing learners with powerful guidance as they explored the AR-based environment. They could engage through not only the senses of sight and hearing but also hands-on activities. Those who were given higher learner control in terms of tool numbers and interactivity considered their visits and learning to be of better quality. They not only were more likely to engage in the museum exhibit but also actively pursued more opportunities for learning. Their active engagement, indicated by the dwelling time and frequent accessing of learning materials, also facilitated their memory and comprehension of the exhibit content.

## Conclusions

In this study, an interactive exhibit using AR technology to provide different levels of learner control was designed and developed for a special specimen collection of a university herbarium. Given the shortage of empirical studies on the learning mechanisms within museum AR applications, this study designed the contextual AR tools for museum learning on the theoretical basis of learner control. A visitor experiment study was conducted in an authentic museum exhibit to investigate how the mechanism of learner control affected visitors’ museum learning. The results clearly indicated that the AR-based interactive exhibit with learner control provided visitors with more volitional choice and resulted in their enriched encoding process and museum experiences. The findings from this study suggested that the affordance of AR technology enhanced the situated feature of the learner control tools such that they blended into the museum environment seamlessly for visitors’ intuitive and effective uses, resulting in visitors’ engagement and learning. The multimodal representations in the AR-based exhibit improved visitors’ coordination of attention with the flow of experience, and the situated control tools provided the visitors with additional metacognitive processing to achieve meaningful interactions with the exhibit content. While all participants achieved medium to high states of flow, those who possessed higher levels of learner control tended to be more engaged in inquiry and in acquiring factual knowledge. This finding echoed those of previous studies using AR in museums, as the visitors tended to become engaged in interactive and concrete exhibit content [46, 61], and it also further verified that learner control could serve as a theoretically sound design framework to enhance interactivity and meaningful learning in the context of museums.

More importantly, given the highly situated nature of museum learning, this study makes a specific contribution to the field research and practices by taking into account both qualitative and quantitative features of learner control in designing the AR control tools and the visitor study. The findings provide empirical support that the visitors valued functionality over quantity of the control tools during their museum visits. They were highly motivated by the contextually appropriate virtual tools, which allowed them to apply real-world schemata to their operation. The visitors viewed the learner control tools not only as a navigation feature but also as an external cognition mechanism to help them process the exhibit content. They used the AR tools more extensively in basic learning tasks, such as in reciting by Magnifier and mnemonics by Notebook. Those who regarded themselves as having lower learner control reported more recital uses of copying materials by Camera or verbatim note-taking in Album and Notes, while those who perceived higher learner control reflected more elaborate uses of summarizing and generative note-taking in Notebook. This finding echoed previous studies on learner control in school settings [16, 20], and further proved that active control was as important and effective in informal educational settings of museums in aiding learners’ acquisition of abstract concepts.

The findings supported the success of integrating the immersive technology of AR and the theoretical framework of learner control to construct museum exhibits. Several practical suggestions for curation are made based on the findings directly available to museum educators, designers and curators. First, when introducing AR technology in museums, it is important to focus on the nature of the immersive experience in addition to the technical features of the simulation technology. Visitors must be required to employ their active perception throughout the AR experiences in order to retain awareness of the physical and virtual worlds, which makes the active learner control even more important. Second, more design considerations should be placed on the qualitative features of learner control, such as the defining features of learner interaction with the exhibit content [17] in this study. Compared to the navigation feature of controlling the sequence and pace of the content, active control of access to the learning resources was regarded by the learners as more critical. Qualitative aspects of learner control can enable curators to include informative cues as physical context [1, 2] that facilitates visitors’ situated learning in museums.

Methodologically, this study contributes to the field studies of learner control by linking learner control with the critical dimensions of museum learning to provide more guidance in exhibit design. In-depth efforts to observe visitor behaviors alone with the analytic investigations through different instruments were made to form a systemic understanding of how the AR affected museum learning from the learner control perspective. However, several limitations of our investigation should be noted. First, the research intention to conduct the visitor experiment in a real museum setting resulted in a relatively small sample size due to the limitations of space and facilities. Therefore, the results should be interpreted with caution. Second, this study conducted a one-off session with voluntary participants. Although their background and experiences were considered in the research design, some participants might have different inherent levels of motivation than the general museum visitors, and some participants might not have had sufficient spare time to achieve flow experiences with the mindset of research participants under the researchers’ observation. Our further work is therefore incorporating separate interactive sessions with ambient observation technologies in order to acquaint visitors with the treatment. So as not to interrupt participants’ visits, this current study adopted qualitative measures of remote observation and interviews. More direct behavioral measurements with physiological devices will be considered and recommended.
